# The environmental effects of anesthetic agents and anesthesia practices

**DOI:** 10.1016/j.jatmed.2024.10.004

**Published:** 2024-12-18

**Authors:** Ramy Khalil, Zhengmin Ma, David Lubarsky, Ke Peng, Fuhai Ji, Hong Liu

**Affiliations:** a California Northstate University, College of Medicine, Elk Grove, CA 95817, USA; b Department of Anesthesiology, First Affiliated Hospital of Soochow University, Suzhou 215000, China; c Institute of Anesthesiology, Soochow University, Suzhou 215000, China; d Department of Anesthesiology and Pain Medicine, University of California Davis Health, Sacramento, CA 95817, USA

**Keywords:** Volatile anesthetics, Halogenated gases, Greenhouse effect, Environment

## Abstract

Anesthesia is a critical domain with considerable capacity to reduce the carbon footprint of healthcare on the environment, particularly in terms of climate change. Volatile anesthetics are halogenated gases that function as potent greenhouse gases and contribute to the greenhouse effect, depletion of the ozone layer, and progression of global warming. However, the use of volatile anesthetics can be decreased significantly through the adoption of more environmentally conscious practices. Regional anesthesia, including intravenous and neuraxial anesthesia, reduces the harmful effects of inhaled anesthetics and has shown to have positive perioperative effects in certain patient populations. Combining total intravenous anesthesia with target-controlled infusion results in optimized infusion rates, thereby reducing both anesthetic use and waste. Techniques such as processed electroencephalogram monitoring can be used to assess the sedation of a patient and prevent over-anesthetizing and consequently decreasing anesthetic use, which may contribute to improved surgical outcomes. Additionally, actively promoting education on low-flow administration practices can enhance the recycling of anesthetic gases and decrease the release of volatile anesthetics into the atmosphere, thereby reducing the carbon footprint of this field. Finally, by refining existing techniques and scavenging systems, xenon, with its physiological stability and minimal environmental impact, could emerge as a leading inhaled anesthetic agent in the future.

## Introduction

The greenhouse effect occurs when compounds, commonly referred to as greenhouse gases (GHGs), trap heat in the Earth’s atmosphere.^1^ Solar energy enters the Earth’s atmosphere and warms the surface; energy from the planet’s surface is then reemitted as infrared energy, which GHGs absorb and reemit back to Earth’s surface.^1^ GHGs are important to maintain a habitable global temperature, but the recent increase in GHG emissions, particularly carbon dioxide (CO_2_), has contributed to the phenomenon of global warming. Amid an environmental crisis, people have been encouraged to reduce carbon outputs by changing daily practices to be more environmentally conscious, such as carpooling to work or conserving electricity. The healthcare sector plays a substantial role in the climate crisis. Take healthcare in the United States as an example, it contributed 7.9 % to carbon emissions nationwide in 2014, totaling about 480 metric tons of CO_2_.^2^ Multiple studies have identified volatile anesthetics as a major contributor to GHG emissions. Corrente and colleagues asserted that nearly half of GHG emissions within healthcare are attributed to hospitals. Operating rooms account for one-third of total hospital waste, and the anesthetic agentscontribute to the depletion of ozone, a compound in the planet’s atmosphere that absorbs harmful ultraviolet (UV) radiation and prevents it from reaching the Earth’s surface.^3^ Beyond environmental impacts, the climate change crisis may also contribute to an increase in vector-borne illnesses, particularly in subtropical climates, presenting challenges to overall health and anesthetic management in affected patients.^4^ In this review, we aim to review volatile anesthetics and their pertinent environmental impacts, while also proposing alternative practices within the operating room to enhance the environmental sustainability of anesthesia delivery.

## Volatile anesthetics and the greenhouse effect

**Fig. 1:**
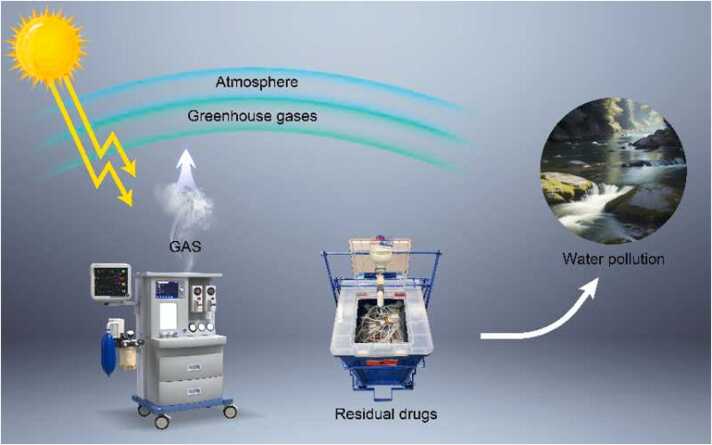
Schematic illustration of gaseous (GAS) and intravenous anesthetic polluting the atmosphere and waterways.

Volatile anesthetics are highly fluorinated gases used to induce and maintain general anesthesia (GA). These compounds, which include nitrous oxide (N_2_O), halothane, isoflurane, enflurane, sevoflurane, and desflurane, can directly contribute to global warming.^5^ Volatile anesthetics are released directly into the environment.^6^ Due to the chemical properties of these compounds, less than five percent of the anesthetic administered during surgery is metabolized by the patient, with the remaining anesthetics being released when patients exhale.^6^, ^7^, ^8^ Exhaled anesthetic is typically scavenged in operating rooms to protect surgical staff; however, it is subsequently released into the atmosphere, where these compounds function as GHGs (Fig. 1).^8^

The global warming potential (GWP) quantifies the environmental effects of anesthetic agents by expressing the agents as CO_2_ equivalents; a higher GWP indicates more heat trapped per unit mass relative to CO_2_ and a more significant contribution to global warming.^5^, ^7^, ^9^ Previous studies have assessed the GWPs of different anesthetics and found that desflurane is one of the greatest contributors as a greenhouse gas, with a GWP of 3700 times that of CO_2_.^5^ One study quantified the contribution of volatile anesthetics to the greenhouse effect in Australia and found that desflurane was responsible for 77 % of the 37,000 tons of CO_2_ equivalents released into the atmosphere over a 7-year period.^7^ Nonetheless, the use of desflurane within the same 7-year period more than doubled, “despite a 9 % inflation-adjusted cost rise”.^7^ A previous study cited the tendency to use desflurane may stem from a misconception that this anesthetic agent reduces the time to extubation.^7^

## Ozone depletion

The ozone layer is an essential component of our planet’s atmospheric stability.^10^, ^11^ Most of the ozone layer is concentrated in the stratosphere, an area of the Earth’s atmosphere which is located above the troposphere, the first layer of the atmosphere.^10^ The most important function of the ozone layer is to prevent ultraviolet radiation B from reaching Earth’s surface.^10^ Depletion of the ozone layer and subsequent temperature increases can expose humans and other species to harmful radiation, potentially contributing to pathologies such as skin cancer, increasing the prevalence of vector-borne illnesses, and ultimately leading to catastrophic effects on our global ecosystems.^4^, ^10^ Upon exposure to UV light, compounds such as chlorofluorocarbons (CFCs) contribute to the destruction of the ozone layer by releasing chlorine or bromine atoms into the atmosphere. These atoms that react with each other and destroy ozone.^11^ Volatile anesthetics are closely related to CFCs and can contribute to ozone depletion through a similar mechanism.^12^, ^13^ However, the anesthetic agents most responsible for ozone depletion is nitric oxide (N_2_O), more commonly known as laughing gas.^13^, ^14^, ^15^ Nitric oxide is recognized as the dominant depleter of ozone in the middle stratosphere, with an atmospheric lifetime of 109 years.^14^, ^15^, ^16^ Therefore, regulating levels of N_2_O utilized during surgery is an essential step in slowing and ultimately halting the progression of global warming.^17^

## Low-flow techniques

Fresh gas flow (FGF), measured in liters (L) per minute, is the volume of gas that is delivered from the anesthetic machine and passes through the breathing system.^18^ Low-flow anesthesia is a technique that involves a decreased fresh gas flow relative to the patient’s alveolar ventilation, which is defined as the volume of air entering/leaving the alveoli per minute.^18^, ^19^ Previous studies have assessed the benefits and risks of low-flow anesthesia in the operating room. Perhaps the most compelling benefit of utilizing low-flow anesthetic delivery is decreasing the release of unmetabolized volatile anesthetics into the environment, resulting in a marked reduction of GHGs.^20^, ^21^ The use of low-flow systems with CO_2_ absorbers is one way to decrease the environmental impact of volatile anesthetics. The low-flow technique also leads to a marked decrease in the amount of volatile anesthetics used during surgery (financially beneficial), especially in a closed circuit system in which the exhaled gas mixture (containing CO_2_ and the volatile anesthetic) passes through an absorber that removes the CO_2_.^18^, ^20^ The remaining volatile anesthetic is then combined with minimal fresh gas flow and is readministered to the patient. Because the volatile anesthetics exhaled by the patient are not vented to the environment, their ability to function as GHGs is markedly diminished. It has been found that low-flow systems can lead to a 60–75 % decrease in the volume of volatile anesthetics utilized to maintain GA, which also translates into a reduction of overall anesthetic cost.^18^ The anesthesia machines used in our institution have a built-in function of monitoring the cost of intraoperative anesthesia agent usage. For example, a 3 % sevoflurane at 2 L/min fresh gas flow costs $ 7.99/hour, while a 0.5 L/min fresh gas flow costs $ 2.00/h at this institution. These savings can be allocated to additional environmental initiatives that may not be self-financing, facilitating hospital administrators' approval of a comprehensive program of environmentally responsible actions.

One phenomenon associated with using low-flow systems is the generation of compound A when sevoflurane passes through CO_2_ absorbers, which contain strong bases like potassium hydroxide (KOH) and sodium hydroxide (NaOH), commonly referred to as soda lime.^20^ When sevoflurane interacts with strong bases, compound A is generated, which has shown nephrotoxic effects in rats. However, despite the administration of hundreds of millions of doses of sevoflurane, no studies have reported analogous toxicities in humans.^20^ Nonetheless, some newer CO_2_ absorbers contain fewer, or even no, strong bases, thereby decreasing the generation of compound A in low-flow sevoflurane administration. Moreover, according to a recent statement from the American Society of Anesthesiologists (ASA), it is recommended to use a 0.5 L/min FGF with sevoflurane, as opposed to the traditionally assumed range of 1 to < 2 L/min.^22^ Educating anesthesia staff about the environmental benefits of utilizing low-flow anesthesia in patients without contraindications can markedly decrease the environmental impact of volatile anesthetics as GHGs, as well as lower the quantity and cost associated with their use.^23^, ^24^

## Processed electroencephalogram (pEEG) monitoring

The pEEG monitors available for perioperative use serve to monitor the depth of sleep/anesthesia. However, unlike ECG, the electrophysiological monitoring of the heart as a standard of care since 1986, there is a reluctance by anesthesiologists to regularly employ EEG, the electrophysiological monitoring of the brain, on a routine basis during everyday surgery.^25^ Intraoperative EEG monitoring has demonstrated the ability to reduce anesthetic exposure. A study found that pEEG-guided anesthesia management reduced propofol delivery by 21 % and the use of volatile anesthetic by 30 % compared to routine care in 921 elderly patients undergoing major noncardiac surgery.^26^ Furthermore, EEG monitoring has led to reduced emergence time after GA. Other studies compared pEEG-guided and non-pEEG-guided anesthetic protocols found that pEEG use decreased the time to extubation and discharge, as well as postoperative nausea and vomiting (PONV), which resulted in better perceived anesthesia quality by patients.^27^, ^28^ By decreasing the use of both inhalational and IV anesthetics, as well as improving perioperative outcomes, the use and attention to intraoperative EEG monitoring constitute an easily implemented technique to mitigate the environmental footprint of anesthetics.

## Other alternatives

### Regional anesthesia

Combining intravenous anesthetics and neuraxial anesthesia, or nerve blocks, has been associated with improved surgical outcomes, including decreased PONV, improved rehabilitation, and increased discharge rates among patients undergoing hip and knee arthroplasties.^5^ Kuvadia and colleagues described that, in addition to positive outcomes among patients, the preferential use of administering regional anesthesia over inhaled anesthetics saved 750 kg of desflurane and 60 kg of nitrous oxide, an equivalent of saving about 2750 gallons of gasoline.^5^ Opting for regional anesthesia, such as nerve blocks, whenever possible rather than inhaled anesthetics can help diminish the harmful environmental effects of these volatile gases.

### Total intravenous anesthesia

Despite their low metabolization rates, volatile anesthetics are consistently used over regional or intravenous anesthetics, which do not function as GHGs and have a much smaller environmental impact. A study showed that, over a 4-year period, only 11 % of hip or knee arthroplasty cases in the United States were performed using regional anesthesia without volatile anesthetic gases.^5^ Increasing the use of total intravenous anesthesia (TIVA) in surgical settings can decrease the use and harmful environmental effects of inhaled volatiles like N_2_O and desflurane.^29^ Intravenous anesthetics such as propofol, ketamine, and etomidate are effective at inducing and maintaining GA with less profound effects on the environment. According to Gaya and colleagues, the GHG effect of propofol is “about four orders of magnitude lower compared with those of volatile anesthetics”, even after considering the environmental effects of drug production and transportation.^30^ Breth-Petersen and colleagues asserted, however, that some anesthesia providers trained during a time when the administration of volatile anesthetics was the standard of practice may feel less comfortable using TIVA than their colleagues who were trained more recently, which may explain their reluctance to adopt this method.^31^ It is important to note that intravenous anesthetics nonetheless have some adverse environmental effects, even if they do not function as potent GHGs. Propofol has been demonstrated to be toxic to aquatic organisms (Fig. 1), especially when not disposed of via the recommended method of incineration.^8^ However, the concentration of propofol that elicits a toxic response in aquatic organisms is many orders of magnitude higher than the predicted environmental concentration of the drug.^32^ Further, the use of TIVA has been shown to increase drug wastage in the operating room.^33^, ^34^ Analyzing procedures performed using TIVA, a study found that the average amount of unused propofol (from 100 mL bottles) was about 30 mL.^34^ By decreasing the size of commonly used drug vials, clinicians can make a positive effort to reduce both drug waste and the overall carbon footprint associated with TIVA.^34^, ^35^ Meanwhile, ensuring that propofol and other intravenous anesthetics are disposed of properly can decrease adverse aquatic effects, making these intravenous anesthetics more environmentally sustainable for long-term use.

### Target controlled infusion

Target-controlled infusion (TCI) of anesthetics is a drug delivery technique that implements computer software to more accurately reach a target concentration based on patient metrics and the pharmacokinetics of the infused drug.^36^ Traditional use of intravenous anesthetics involves the administration of either a large-dose bolus or continuous infusion; however, these methods of administration are confounded by the accumulation of the drug in the patient’s tissues.^36^, ^37^ TCI addresses this problem by adjusting the infusion rate of the anesthetic agents based on the predicted bioaccumulation of the drug.^36^, ^37^ Using TCI with TIVA can significantly reduce the use of both volatile and intravenous anesthetics. Opting for TIVA decreases volatile anesthetic use, and administering TIVA via TCI results in less anesthetic consumption due to the continuous infusion and accurate dosing. Comparing anesthesia with propofol TCI to sevoflurane, it has been found that fewer interventions to adjust anesthetic depth were necessary with TCI. Moreover, patient satisfaction with the anesthesia experience and time to extubation were similar in both groups.^38^

### Xenon

Xenon is an inert noble gas naturally present within our atmosphere at very low concentrations (0.000008 % of the atmosphere).^39^ Previous studies have highlighted the chemical and physical properties of xenon that make it a suitable candidate as an alternative anesthetic agent. Some of these properties include low reactivity and nontoxicity, as well as the fact that xenon is nonflammable, nonexplosive, and nonteratogenic.^40^ Initial experiments with xenon in the 1940s demonstrated its analgesic effects in mice, and subsequent studies successfully achieved a state of full anesthesia using the compound.^39^ One advantage of xenon as an anesthetic agent is its potency compared to nitrous oxide, and xenon has a minimum alveolar concentration of 71 %, whereas nitrous oxide has a MAC of 104 %.^39^, ^41^ Therefore, xenon is a more potent anesthetic agent, requiring a smaller dose to achieve therapeutic sedation. In addition, xenon possesses a lower blood-gas partition coefficient compared to nitrous oxide, allowing it to induce and facilitate anesthesia emergence more rapidly.^39^ Furthermore, xenon is 1.5 times more potent as an analgesic than nitrous oxide, likely due to its observed effects in the dorsal horn of the spinal cord. Additionally, it is very effective at preserving cardiovascular stability during surgery.^41^, ^42^ Xenon did not have any major effects on the chemical or electrical conducting systems in animal study, further supporting its potential use as an anesthetic agent.^41^ Environmental effects of xenon as an anesthetic agent include its inability to act as a pollutant when being released into the atmosphere.^40^ Unlike the volatile anesthetics in current use, xenon does not function as a GHG and therefore does not contribute to global warming.^40^, ^41^ A likely reason xenon has not yet become a widely used anesthetic agent, despite its benefits, is its high cost.^39^, ^40^, ^41^ A complex procedure involving the distillation of atmospheric air is necessary to extract xenon, leading to high production costs.^40^ While nitrous oxide costs only a fraction of a dollar per liter, the price of xenon may vary between about 10–20 dollars per liter.^39^, ^40^, ^41^ However, the relatively high cost of xenon may be offset by using methods such as low-flow anesthesia to decrease the total amount of anesthetics utilized.^40^, ^41^ Xenon recycling units would have to be implemented as well to make this gas a more cost-effective anesthetic alternative.

## Conclusions

Anesthesia is a critical domain with considerable capacity to reduce the carbon footprint that healthcare has on the environment, particularly in terms of climate change. Volatile anesthetics are halogenated gases that function as potent GHGs and contribute to the greenhouse effect, depletion of the ozone layer, and progression of global warming. However, the usage of volatile anesthetics can be decreased significantly through the adaption of current anesthesia practices to be more environmentally conscious. Regional anesthesia reduces the harmful effects of inhaled anesthetics and has shown to have positive perioperative effects in certain patient populations. Combining TIVA with TCI results in optimized infusion rates, thereby reducing both anesthetic usage and waste. Techniques such as pEEG monitoring can be used to assess the sedation of a patient and prevent over-anesthetizing, and consequently decreasing anesthetic usage and leading to better surgical outcomes. Furthermore, actively increasing education to raise the awareness of the greenhouse effect caused by volatile anesthetics is a means of reducing the carbon footprint in this field. Finally, by refining existing techniques and scavenging systems, xenon, with its physiological stability and minimal environmental impact, could emerge as a leading inhaled anesthetic agent in the future.

## CRediT authorship contribution statement

**Ke Peng:** Writing – review & editing, Visualization, Validation, Resources, Conceptualization. **Fuhai Ji:** Writing – review & editing, Visualization, Validation, Resources, Conceptualization. **Hong Liu:** Writing – review & editing, Validation, Supervision, Resources, Project administration, Methodology, Funding acquisition, Formal analysis, Conceptualization. **Ramy Khalil:** Writing – review & editing, Writing – original draft, Visualization, Formal analysis, Data curation. **Zhengmin Ma:** Writing – review & editing, Writing – original draft, Visualization, Resources, Formal analysis, Data curation. **David Lubarsky:** Writing – review & editing, Validation, Supervision, Resources, Conceptualization. All authors have read and agreed to the published version of the manuscript.

## Disclosure statement

Not applicable.

## Ethical statement

Not applicable.

## Funding

This work was supported in part by the Department of Anesthesiology and Pain Medicine, University of California Davis Health and NIH grant UL1 TR000002 of the University of California Davis Health.

## Declaration of competing interest

The authors declare that they have no known competing financial interests or personal relationships that could have appeared to influence the work reported in this paper. Fuhai Ji is an Associate Editor for Journal of Anesthesia and Translational Medicine and was not involved in the editorial review or the decision to publish this article.

## Data Availability

All study data are included in the article.
